# Transforming social norms to improve girl-child health and well-being: a realist evaluation of the Girls’ Holistic Development program in rural Senegal

**DOI:** 10.1186/s12978-021-01295-5

**Published:** 2021-12-03

**Authors:** Anjalee Kohli, Bryan Shaw, Mathilde Guntzberger, Judi Aubel, Mamadou Coulibaly, Susan Igras

**Affiliations:** 1 Center for Child and Human Development, Georgetown University, Washington, DC USA; 2 Proteknôn Consulting Group, Paris, France; 3 The Grandmother Project: Change Through Culture, Mbour, Senegal

**Keywords:** Social norms, Reproductive health, Communication, Empowerment, Education

## Abstract

**Background:**

Early adolescence is a critical period where social norms, attitudes, and behaviors around gender equality form. Social norms influence adolescent choices and behaviors and are reinforced by caregivers and community members, affecting girls’ reproductive health and educational opportunities. Understanding how to shift these often-interconnected norms to delay child marriage, pregnancy and keep girls in school requires understanding of the structure and dynamics of family and community systems. The Senegalese and American non-governmental organization, the Grandmothers Project—Change through Culture, seeks to address these intertwined factors through innovative community change strategies that build on the specific structure and values of West African collectivist cultures.

**Methods:**

The Girls’ Holistic Development approach in rural Vélingara, Senegal posits that by increasing recognition, knowledge and empowerment of elder community women and reinforcing intergenerational communication and decision-making, community members including girls will support and advocate on behalf of girls’ interests and desires. We assessed the Girls Holistic Development approach using Realist Evaluation with a mixed-method, quasi-experimental design with a comparison population. We examined differences in intergenerational communication, decision-making and descriptive and injunctive norms related to early marriage, pregnancy and schooling.

**Results:**

After 18 months, intergenerational communication was more likely, grandmothers felt more valued in their communities, adolescent girls felt more supported with improved agency, and norms were shifting to support delayed marriage and pregnancy and keeping girls in school. Grandmothers in intervention villages were statistically significantly more likely to be perceived as influential decision-makers by both VYA girls and caregivers for marriage and schooling decisions compared to girls and caregivers in comparison villages.

**Conclusions:**

This realist evaluation demonstrated shift in social norms, particularly for VYA girls, in intervention villages favoring delaying girls’ marriage, preventing early pregnancy and keeping girls in school along with increased support for and action by grandmothers to support girls and their well-being related to these same outcomes. These shifts represent greater community social cohesion on girl-child issues. This research helps explain the linkage between social norms and girls’ reproductive health and education outcomes and demonstrates that normative shifts can lead to behavior change via collective community action mechanisms.

## Plain language summary

During adolescence in Senegal, as elsewhere, decisions on whether to keep girls in school and at what age to marry girls are made by their caregivers and influenced by family and community members. Early pregnancy occurs at these ages, either before or during marriage. These social influences, called social norms, set expectations for parents and girls.

The Grandmothers Project—Change through Culture developed an intervention to shift social norms and change these three outcomes—early pregnancy, early marriage and keeping girls in school. The project, called Girls Holistic Development (GHD), builds on local relationships between girls, grandmothers, parents and community leaders and local values to facilitate discussion, reflection, collaboration and advocacy.

This study used realist evaluation methods, including qualitative and quantitative interview and focus group discussions, to understand whether these shifts in norms and behaviors took place. Research took place with girls, grandmothers and male and female caregivers 18 months after GHD started. Quantitative survey included 7 intervention and 7 comparison villages.

Results supported GHDs’ expectations and strategy. In intervention villages, grandmothers and girls reported closer relationships; parents considered grandmothers important sources of advice. Girls, grandmothers and caregivers described social expectations as favoring girl’s education, marriage at older ages and development of strategies to prevent girl’s pregnancy in intervention villages.

This evaluation provided strong support for GHDs’ ability to shift social norms to improve girls’ outcomes. By working with local relationships and values, GHD created more communication between community and family members and facilitated increased social bonds within the community.

## Background

Early adolescence is a critical period where boys and girls experience physical, social, mental, and emotional development [1], and where social norms, attitudes, and behaviors around gender equality form. Very young adolescents (VYA) (aged 10–14 years) make up 8% of the global population [2]. VYA girls, especially those living in rural areas or growing up poor in developing countries, are vulnerable to child marriage and adolescent pregnancy, each of which affects girls’ school education and health [2]. Such adolescent realities are influenced by belief systems, power structures, social norms, and other factors. Social norms, the implicit, informal rules that most people accept and follow influence behavior and can protect or reinforce gender (in)equalities [2, 3]. To improve VYA well-being at scale, a deeper understanding is needed of which interventions improve VYA outcomes and create an enabling environment, including understanding how interventions achieve change. We present findings on three primary outcomes (early marriage, adolescent pregnancy, retention in school) and pathways to change from a mixed-methods evaluation of an innovative community-driven project grounded in collectivist socio-cultural values in Senegal, the Girls Holistic Development (GHD).

Adolescent marriage, pregnancy, and girls’ education represent a nexus whose interactions are not easy to separate. All three domains influence a girl’s life trajectory including her health, economic opportunity, and the lives of her children. Girls’ education, for example, is proven to have wide-ranging benefits, including reducing child marriage, adolescent pregnancy, gender-based violence (GBV) and child mortality and promoting gender equality and health [4–6].

Child marriage is embedded within social and familial structures in West Africa [7, 8]. In Senegal, the minimum legal age of marriage is 16 for girls and 18 for boys. In practice, local communities and families determine when girls get married [9]. This is particularly true in the Kolda Region where 68% of girls are married before 18 years, more than twice the national prevalence (31%) [10]. In Senegal also, adolescent pregnancy is more often the result of child marriage [11].

While education in Senegal is compulsory and free up to age 16, in practice, social norms and other factors determine whether girls remain in school [12]. Many parents depend on subsistence agriculture with family labor to survive. Those with limited resources often choose to send their sons to school over their daughters [13]. Primary-to-secondary school transitions show almost half of girls leaving school after primary education: 56% of girls attend primary school and only 27% attend secondary school [2]. Among other reasons cited globally for girls leaving school (e.g., menstrual hygiene management, sexual and reproductive health, violence), child marriage is cited as a main reason for girls leaving school [2, 14].

Understanding how to encourage later marriage, later child birth and girls’ retention in school necessitates in-depth understanding of individual and collective behavioral drivers and their interconnections. Our scoping review of published research on girl-children in Senegal in the last decade indicates that few interventions holistically look at the influence of social factors such as intergenerational communication, community social cohesion, and collective action on these outcomes. The Senegalese and American non-governmental organization, the Grandmothers Project—Change through Culture (GMP), seeks to address these intertwined factors through innovative community change strategies that build on the specific structure and values of West African collectivist cultures.

### Intervention description and theory of change

In rural Vélingara Department where GHD operates, family and community systems are characterized by age, generational and gender hierarchies. Decision-making related to girls’ education and marriage is collective, with adults and elders usually playing leading roles; and little space is given to girls’ own opinions. Parents have authority over adolescents’ mobility, social interactions, and decisions. According to tradition, grandmothers and aunts have primary responsibility for girls’ socialization, but today their role and influence has diminished [15]. The wider breakdown in communication between generations and the limited communication between sexes are barriers to communication and decision-making for adolescent girls.

GHD uses participatory, dialogical approaches to build relationships and community consensus on girl-child issues. Over a 2 to 3-year period, GHD staff organize a series of community forums each year that involve adolescents, parents, elders, traditional and religious leaders, and teachers. Strategically focusing discussions across three generations (elders, parents, adolescents), between sexes within communities, and between community leaders. Participants in the different meetings discuss socio-cultural-religious expectations relating to girls’ education, child marriage, adolescent pregnancy, and female genital cutting. GHD concurrently promotes grandmother–girl relationships to reinvigorate, update, and expand grandmothers’ traditional roles as counselors on girl-child issues and give girls’ greater voice on their issues. These dialogue-based activities strengthen relationships, social cohesion, and communication between generations and sexes. The approach empowers girls and grandmothers to speak up and advocate their positions, and concurrently builds an enabling social environment where family and community actors’ support change for adolescent girls.

**Fig. 1:**
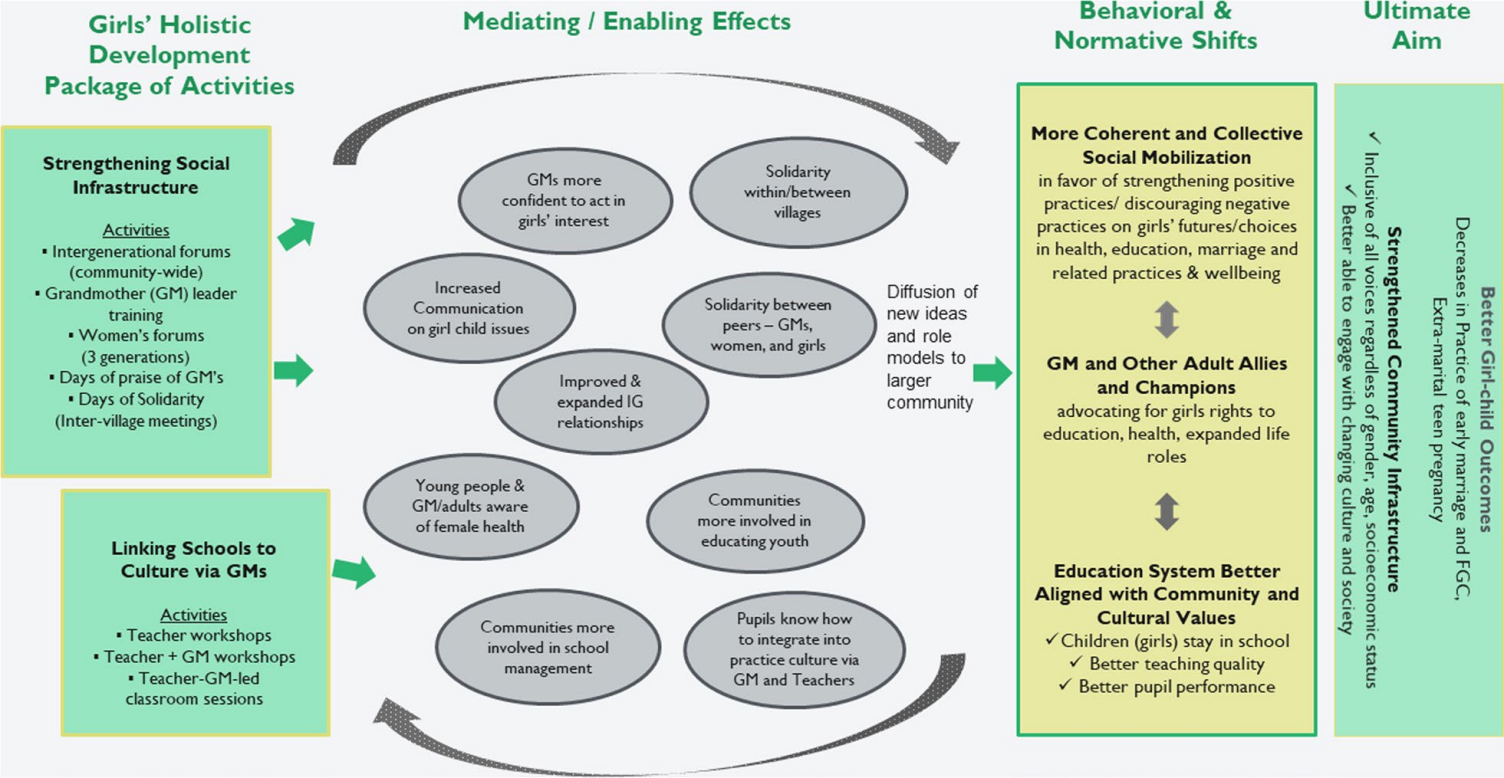
Girls’ Holistic Development program theory of change (2016)

Based on multiple GHD action-research studies, GHD has a good understanding of implementation processes and community level effects [16]. GMP and the Institute for Reproductive Health (IRH) at Georgetown University established a partnership, as part of the USAID-funded Passages Project (2016–2018), to conduct a Realist Evaluation to use the program theory of change, mixed-methods research and program data to understand whether and how the GHD intervention achieves change [17]. A Realist Evaluation uses the program’s theory of change to understand and guide testing on how project activities operating in a certain context lead to a series of intermediate changes or mediating effects that eventually lead to project outcomes. The approach is well suited to norms-shifting interventions operating in complex community settings as it allows deconstruction and testing of change pathways leading to expected program outcomes [18, 19]. The GHD theory of change (ToC) (Fig. 1) that guides this impact evaluation was developed in 2016 with GMP staff and stakeholders using a participatory approach to make explicit the anticipated pathways of change. The ToC shows how GHD activities lead to intermediate effects, including increased agency of girls, women and grandmothers. It posits that through the dialogical process, not only the role of and respect for elders is restored, but grandmothers are empowered to establish a trusting relationship with girls and advocate on their behalf and communities are led to find alternative solutions, for example, to address girls’ early departure from school [20]. Over time, these individual and collective actions that support adolescent girls contribute to shifts in the normative environment, for example, what a community believes is the acceptable path for young girls to adulthood. The realist evaluation of GHD included development of a program theory of change, review of existing action-research reports and conducting several rapid studies to test change pathways, and an impact study. This paper shares findings from the quantitative and qualitative research to examine program pathways of change to understand program impact on adolescent outcomes on child marriage, adolescent pregnancy and girls’ education and the achievements in shifting social norms, enhancing social cohesion and communication.

## Methods

GHD expanded its approach to a new intervention area in the Kolda region offering an opportunity to do a quasi-experimental evaluation of effects and outcomes after 18 months of implementation. The study, which employed post-test only, control group design, was well-suited to the evaluation [21] given already-developed understanding of GHD processes and effects from earlier action-research studies [16]. There were no other community-based programs addressing girl-child outcomes in the area (Mamadou Coulibaly, personal communication).

The quantitative endline study took place between January and March 2019 in seven intervention villages and seven comparison villages, selected on similar characteristics to intervention sites, e.g., ethnicity, village size, and the absence of other NGO interventions. The qualitative study occurred in September 2018 in four of the seven intervention villages. IRH partnered with faculty from the Université Cheikh Anta Diop de Dakar to conduct the evaluation.

### Participant sample

#### Cross-sectional survey

The study sample included 399 mostly unmarried VYA girls, 196 grandmothers, and 205 caregivers from seven villages in the Némataba Commune that received the full GHD intervention and seven non-intervention villages were purposively selected for comparison. Due to the small size of the seven intervention villages (between 100 and 450 total residents), data collectors approached every eligible adolescent girl and grandmother in GHD and comparison villages. Every other adolescent girl interviewed was asked to nominate the individual they considered their primary adult caregiver, creating the sample of caregivers. Girls were eligible if between the ages of 10–14 years at the beginning GHD intervention activities (March 2017). In this setting, a respected, elder woman (of an age where most women have biological grandchildren) is considered a grandmother in her community regardless of whether she had grandchildren. Grandmothers were identified by village leaders and approached for the study. Less than 1% of individuals approached (3 VYA girls, 2 grandmothers, and 3 caregivers) declined to participate in the survey in both intervention and comparison villages.

#### Focus group discussions (FGD) and in-depth interviews (IDI)

A subset of participants in GHD were included in the qualitative study. Four participants of each type (adolescent girls, caregivers, grandmothers) participated in IDI in each of the four villages. Five FGD were held in each of the four villages with adolescent girls, grandmother leaders, mothers and fathers and community leaders. All participants were purposively selected with village leaders’ and GHD support. Village leaders from intervention villages also participated in a separate FGD.

### Measures

#### Quantitative data

Questionnaires for the three study groups included questions on socio-demographics, intergenerational communication and support, individual attitudes and self-efficacy, actual/intended behaviors and social norms related to early marriage and school retention among adolescent girls (Fig. 1). Interviewers used tablets (ODK platform) during face-to-face interviews with VYA girls, caregivers, and grandmothers in intervention and comparison villages.

Previously established measures [22–25] and in particular, theory emphasizing social norms as “social beliefs” [26] informed identification of two types of social norms that were assessed in this study: (1) one’s perception about typical behaviors of their reference groups, or descriptive norms; and (2) one’s perception about (dis)approved behaviors of their reference groups, or injunctive norms. Reference groups are those whose actions and opinions matter to an individual and important in upholding normative beliefs [23, 24].

**Fig. 2:**
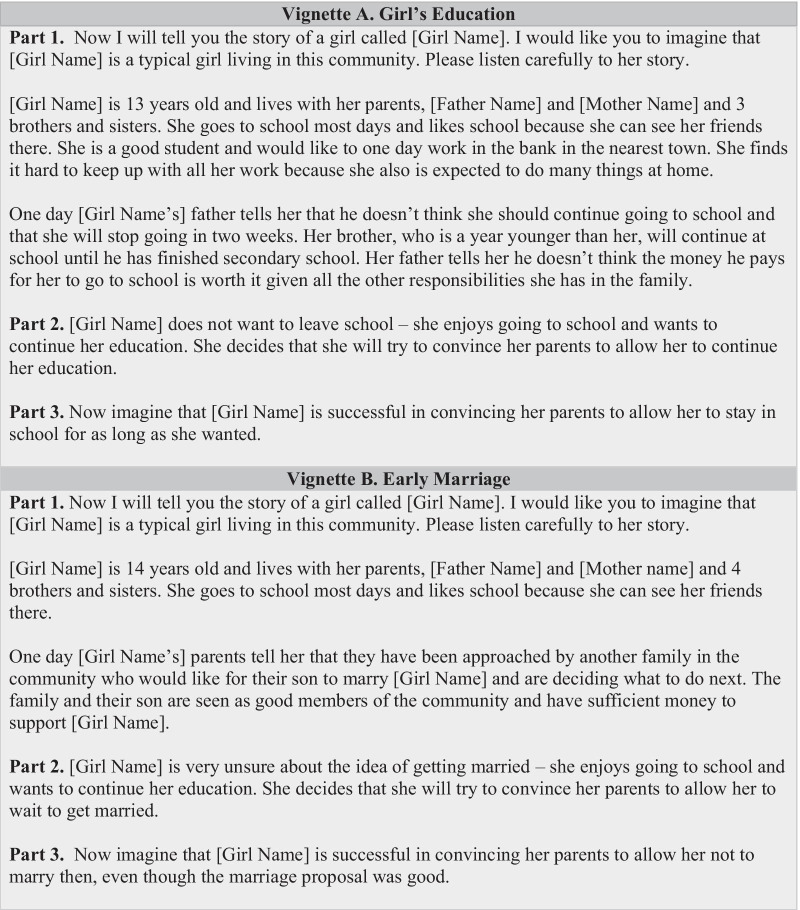
Sample vignettes used in quantitative surveys

A pre-survey social norms assessment [16, 27] and past action-research studies by GMP identified norms and reference groups relevant to the behaviors of interest and informed the social norms survey questions. For example, adolescent girls, caregiver, and grandmothers answered questions on their perceptions of a typical behavior such as early marriage, approved behavior of primary reference groups, and expectations of sanctions to delay marriage. Culturally-appropriate stories in the form of social norms-focused vignettes [24, 28–30] explored norms and behaviors in the community (Fig. 2). Vignettes included questions about reference groups, descriptive norms, injunctive norms, and potential sanctions for behaviors that deviate from the social norm.

#### Qualitative data

IDI and FGD guides explored how the GHD program contributed to change in relationships, communication, and decision-making across ages and genders, including their effects on the outcome behaviors. Semi-structured IDI included questions about participation in the intervention, grandmother roles and relationships and used a social norms-focused vignette to understand more about self-efficacy. The FGDs asked similar questions on participation and experience with the intervention, and communication and advice giving networks for adolescent girls. The 20 FGDs and 48 IDIs were conducted in Pulaar, audio-recorded and transcribed into French by the interviewer. A second interviewer checked the accuracy of each transcription.

### Data management and analysis

Quantitative and qualitative data were first analyzed separately, as described below. Following this, the IRH team held an internal workshop to review and compare findings, map them on to the ToC and examine whether and how the data provide support to the hypothesized pathways of change.

#### Quantitative data

Statistical analyses were conducted in Stata 16.0 (StataCorp. 2019. *Stata Statistical Software.* College Station, TX). Descriptive statistics were presented for all variables. Chi-squared tests for significant differences were performed on key outcome variables. This study adopted an intention-to-treat approach to analysis, with respondents assigned to intervention and comparison villages regardless of reported exposure to the program. Differences were assessed between intervention and comparison populations, by levels of self-reported engagement with the intervention and caregivers by gender. Attitudinal and normative questions used 4-point Likert scales (i.e., none, some, many, or most of their reference group engaging in or (dis)approving of an action). However, given low variation in Likert response options, these questions are reported as dichotomous variables (i.e., none/some or many/most).

#### Qualitative data

NVivo 10 (QSR International. 2014. *NVivo Qualitative Analysis Software.* London, UK) was used for analysis. Content analysis mostly followed the study research questions and intermediate outcomes in the GHD theory of change. In addition to exploring pathways of change, analysis sought to describe between-group comparisons. A subset of transcripts was initially read in full to identify initial themes and codes, which led to coding and analysis around four main themes: project strategies, changes in norms and practices, individual change (belief, knowledge, capacity), and collective change.

## Results

After describing survey participant characteristics, we focus on three intermediate effects on the GHD pathway to change (Fig. 1): development and strengthening of grandmother–adolescent relationships, grandmothers as change agents for VYA girls’ health and well-being, and intergenerational relationships and dialogue. As results will demonstrate, these three pathways provide the foundation for behavior and normative changes at individual and community level, followed by behavioral and normative outcomes.

### Socio-demographic characteristics of survey participants

Almost all participants were from the Pulaar ethnic group and practiced Islam (Table 1). VYA girls participating in the GHD intervention and study ranged in age from 12 to 16 years. Despite national laws against marriage before the age of 16, 2.8% of VYA girls reported that they were currently married. Nearly two-thirds were attending or had completed primary school (66.1%) and roughly one-third (30.1%) were attending secondary school. Primary caregivers were evenly split between men and women, having a mean age of 45. Most were married (92.2%) with few reporting secondary school attendance or higher (5.8%). Grandmothers had a mean age of 61, most were married (60.2%) or widowed (38.3%), and few had any formal education (5.6%). No statistically significant differences were observed for socio-demographic variables comparing respondents from intervention and comparison villages.

**Table 1:** Socio-demographic characteristics of very young adolescent girl, caregiver, and grandmother survey respondents in intervention and comparison areas of Némataba Commune, Senegal, 2019

	VYA girls (%)		Caregivers (%)		Grandmothers (%)	
	Int.	Comp.	Int.	Comp.	Int.	Comp.
Total (n)	161	238	80	125	56	140
Mean age in years (SD)	13.9 (1.4)	13.9 (1.5)	46.7 (12.6)	43.6 (11.2)	62.0 (13.3)	60.0 (13.1)
Sex						
Female	100.0	100.0	53.8	52.0	100.0	100.0
Male	–	–	46.2	48.0	–	–
Ethnicity						
Pulaar	97.0	97.5	92.5	92.0	98.2	94.2
Religion						
Muslim	100.0	99.6	100.0	97.6	100.0	99.3
Marital status						
Married	2.5	2.9	91.1	92.8	50.0	64.3
Engaged	15.5	12.6	0.0	0.0	0.0	0.0
Single/divorced	82.0	84.5	1.3	2.4	0.0	2.1
Widowed	0.0	0.0	7.6	4.8	50.0	33.6
Educational attainment						
None	3.1	4.2	75.0	78.4	87.5	91.1
Some/completed primary	64.6	67.3	15.0	18.4	12.5	8.2
Some secondary or higher	32.3	28.6	10.0	3.2	0.0	0.7
Socioeconomic tertiles						
Poorest	36.0	34.1	35.0	40.0	39.3	36.4
Middle	24.8	35.8	31.3	31.2	23.2	22.2
Wealthiest	39.1	30.1	33.8	28.8	37.5	41.4

No statistically significant differences observed between respondents from intervention and comparison villages

### Exposure to GHD activities

In intervention villages, 80.1% of adolescent girls, 76.3% of caregivers, and 85.1% of grandmothers reported attending at least one GHD intervention activity. As expected, reported exposure to GHD activities in comparison villages was very low; less than 4% of each of the three target groups reported attending at least one GHD intervention activity.

### Intermediate effects seen in change pathways

#### Development and strengthening of adolescent–grandmother relationships

The qualitative assessment described grandmothers as previously having lost social status and regaining connections with their families and communities through the inter-generational communication activities of the project in recent years. During IDIs and FGDs, adolescents, caregivers, and grandmothers explored before-after status and relationship changes due to the GHD intervention.

From a grandmother in Bakayoko: “Grandmothers were seen as witches, and young girls did not want to be near them. Daughters-in-law did not want to care for grandmothers…but now this has changed. Now you can hold and take care of your grandchild, spend the night and share food with them”

An adolescent girl from Koumera described new and fun relationships and wanting to spend time with grandmothers, “grandmothers should approach their grandchildren on their knees, tell them jokes and stories, and teach them about community traditions.”

A father in Nemataba described grandmothers as engaging in play to build trust with girls and to create a space for listening and advising them, a relationship their mothers do not have, “girls say that they feel free…their communication is good. They (grandmothers) joke and advise children at the same time. There are many things that mothers cannot say to adolescents, but grandmothers can speak about these things.”

#### Grandmothers as change agents for VYA girls’ health and well-being

The GHD ToC proposes that with increased respect and recognition of grandmothers in families and communities and trusting relationships between grandmothers and girls, grandmothers will support and advocate on behalf of girls’ interests and desires. Survey findings indicate this occurred (Table 2). Grandmothers in intervention villages were statistically significantly more likely to be perceived as influential decision-makers by both VYA girls and caregivers for marriage and schooling decisions (approximately one-quarter of respondents for each group) compared to girls and caregivers in comparison villages (less than 10%).

**Table 2:** VYA girls’ and caregivers’ perceptions of grandmothers as decision-makers and supporters of girls’ well-being in Némataba Commune, Senegal, 2019

	VYA girls (%)		Caregivers (%)	
	Intervention	Comparison	Intervention	Comparison
	*N* = 161	*N* = 238	*N* = 80	*N* = 125
Perceived that grandmother would be/was a major decision-maker with regards to:				
Marriage decisions	27.3***	1.3***	30.0**	16.8**
Schooling decisions	24.8***	2.1***	16.3**	6.4**
In past year, talked with/asked advice and support from a grandmother about:				
Marriage	40.4***	10.1***	87.5	80.0
Schooling	52.2***	21.9***	73.8***	45.6***

**Statistically significant difference at p < 0.05

***Statistically significant difference at p < 0.01

At the same time, girls, caregivers, and communities accorded more respect and recognition to grandmothers. These attitudinal and belief shifts led to bi-directional *actions* such as girls seeking support and grandmothers advocating on their behalf.

A girl from Bakayoko: “if our parents want to marry us early, grandmothers will not accept this...That is why we go speak to our grandmothers when our parents want to marry us. Our fathers are scared of their mothers…when grandmothers speak to our fathers, our fathers stop pursuing our marriage.”

A father from Bagayoko validated this grandmother role, “they are elders. They guide us to the right path...we have to obey them.”

Grandmothers themselves affirmed these shifts in advocating for young girls (Table 3). Grandmothers in intervention villages were more likely to report that they would support a VYA girl or their caregivers to delay marriage (69.6%) compared those in comparison villages (59.7%). However, we did not see significant differences in grandmothers’ willingness to support a girl or her caregiver to stay in school. Likewise, grandmothers in intervention villages were more likely to report that they perceived that they could influence key decision-makers to delay marriage for a young girl (69.6%) compared to grandmothers in comparison villages (42.9%). Again, there was no significant difference for a grandmothers’ perception that she could advocate to keep a girl in school between intervention and comparison villages.

**Table 3:** Grandmothers’ perceptions of their support for VYA girls’ well-being in Némataba Commune, Senegal, 2019

	Intervention (%)	Comparison (%)
	*N* = 56	*N* = 140
Perceive that adolescent girls would come to me for advice/support if:		
Future husbands/families are pressuring them to get married < 16 yrs	76.8***	30.2***
Parents are considering removing girls from school	85.7***	39.3***
Perceive that caregivers of adolescent girls would come to me for advice/support if:		
They are considering marriage for daughter < 16 yrs	69.6***	27.9***
They are considering removing daughter from school	73.2***	43.1***
In general, I feel that caregivers of adolescent girls are willing to come to me for advice and support	80.4***	39.3***
In general, I feel that I am a valued part of this community	87.5***	60.4***
If VYA girl/her parents came to you for your support, you would support them to:		
Delay marriage	69.6**	59.7**
Keep girls in school	83.9	84.2
Perceives that grandmother could influence decision-makers to:		
Delay marriage	69.6***	42.9***
Keep girls in school	67.9	59.3

**Statistically significant difference at p < 0.05

***Statistically significant difference at p < 0.01

#### Intergenerational relationships and dialogue

GHD seeks to build community social cohesion through mutually-reinforcing dialogical activities such as intergenerational forums, women’s forums, and days of solidarity. Such activities increase communication across generations on adolescent girls’ lives and well-being, improve understanding of women’s health issues, and increase inter-and intra-group solidarity.

Participants described how creating public space for conversation promoted a shift in gender attitudes and actions. For example, fathers now get involved in their daughter’s education, and communication improved between fathers and mothers.

One mother from Sare Yire: “communication is more developed especially between women and their husbands. Before the head of household (male) made all the decisions, but this has changed.”

A father from Sare Yire: “Before the project, the parents did as they saw fit with their daughters, but once the project came ... even if we are in a hurry to give girls in school away in marriage, we must let them study because studies are useful”.

A father from Sare Yire: “we have understood that we are equal because mothers and fathers are the same.”

This suggests that at 18 months into intervention, GHD fostered better comprehension and cohesion between men and women, including understanding their shared goals and responsibilities, a foundation for gender equity pathways. The intervention period continued beyond the 18-months timeframe when this evaluation was conducted, indicating potential to shift gender roles and responsibilities or household hierarchies.

Survey findings (Tables 2 and 3) point to such shifts, reflecting statistically-significant communication and intergenerational relationship differences between intervention and comparison groups. Adolescent girls in intervention villages were more likely to report seeking advice or support from a grandmother about marriage (40.4%) and schooling (52.2%) compared to girls in comparison villages (10.1% and 21.9%, respectively). According to grandmothers themselves, those in intervention villages perceived that adolescent girls would seek their support when they experienced pressure to marry before the age of 16 (76.8% vs. 30.2%) and when parents were considering removing a girl from school who wanted to continue schooling (85.7% vs. 39.3%). Evidence of caregivers’ strengthened intergenerational bonds in intervention villages described reliance on grandmothers to advise and support them on decisions about their daughters’ education (73.8%) compared to caregivers in comparison villages (45.6%). Interestingly, grandmothers’ roles in providing marriage advice for caregivers are high and similar in both intervention (87.5%) and comparison (80.0%) villages.

In general, grandmothers in intervention villages were more likely to perceive that caregivers come to them for advice and support (80.4% vs. 39.3%) and were more likely to report that they felt a valued member of their community (87.5% vs. 60.4%). Evidence of strengthened collective and more equitable actions was also seen. Participants described a revitalization of community responsibility for the education of children.

A grandmother in Bagayako: “before you could not educate or correct just anyone’s child and they could not educate or correct yours. Now, the entire village has the right to correct and educate the children of the village.”

Participants reported that previously fathers made decisions for girls with limited if any, discussion. GHD’s intergenerational dialogue and grandmother-facilitated community dialogues gave voices to all to express their opinion even when it contradicts a father’s choice.

A mother from Sare Yire: “before the project, if we wanted to marry our daughter, we just did it. Fathers would give their daughter in marriage without knowing her preferences….when the son of a known person visited the family to say he likes your daughter. The father did not tell the young man to ask his daughter if she likes him. He would say, this man came and proposed to my daughter, and we reached an agreement…”

Through activities that engage adolescents and adults, building social cohesion and ensuring girls and grandmother voices are heard and valued, adults became more aware of VYA experiences, hopes, and concerns.

A grandmother in Koumera described growing adolescent agency as pushing for change; they are *“not accepting to be in the back. They all want to be in the front.”* Another said, *“adolescent girls have the courage to speak and express their feelings.”*

### Social norms and behavioral outcomes

GHD posits that the above activities and effects foster normative shifts that contribute to behavioral change. While a study period reflecting only 18 months of a change process might lead to normative shifts, behavioral changes may not (yet) have been measurable at the time of the assessment. Indeed, while changes in behavior were not evident, some normative expectations did change, particularly for VYA girls in regards early marriage and girls’ education, and are presented below.

#### Shifts in normative expectations to delay early marriage

Caregivers in intervention villages were statistically less likely to report that they intend to marry their VYA daughter before the age of 16 (13.3%) than caregivers in comparison villages (27.9%). The quotes below reflect change in decision-making dynamics in the family and community that will favor girl-outcomes in the future.

From a mother in an FGD in an unnamed village: *“We refuse! Listen to me! When my daughter was to be married, and her husband came to take her to his house, I said, ‘No!’ Did she leave? No! We said that until she finishes her schooling, she cannot leave our house. And she did not leave.”*

From a community leader in an FGD*: A husband came this year to me to say he was marrying [his daughter], and I told him that the girl was still studying… You can go home! And he left.”*

Similarly, the social norms vignette, which described a VYA girl’s family receiving a marriage proposal from a family of good standing, indicated statistically significant shifts (p < 0.05 or less) in normative expectations between intervention and comparison villages (Table 4). VYA girls in intervention villages were statistically more likely to perceive that VYA girls’ caregivers would disapprove of such a marriage before 16 (65.2%) than girls in comparison villages (52.4%). VYA girls were statistically more likely to perceive that their VYA peers would disapprove of such a marriage before 16 (93.8%) than VYA girls in comparison villages (88.7%). Although moving in the desired direction, not all changes were significant: more caregivers and grandmothers in intervention villages perceived that caregivers would not approve the marriage, but the differences were not statistically significant. The perception of the number of unmarried VYAs in the community (descriptive norm) was high in intervention villages, but not significantly different to comparison populations. Likewise, we did not see significant differences in expectations of sanctions (injunctive norm) for delaying a “good marriage” between girls in intervention and comparison villages. In all normative domains, grandmothers in intervention and comparison groups held similar views as those in comparison groups. Finally, in qualitative study, early marriage was closely tied with adolescent pregnancy where parents used early marriage as a means to prevent or respond to girls’ pregnancy. Therefore, norms already considered pregnancy unacceptable with actions such as early marriage employed, at times, to prevent stigma and other consequences.

**Table 4:** Normative outcomes for VYA girls, caregivers, and grandmothers in Némataba Commune, Senegal, 2019

	VYA girls		Caregivers		Grandmothers	
	Intervention (%)	Comparison (%)	Intervention (%)	Comparison (%)	Intervention (%)	Comparison (%)
	*N* = 161	*N* = 238	*N* = 80	*N* = 125	*N* = 56	*N* = 140
**Early marriage**						
*Perceive that* ___ *in respondent’s community*						
Girls < 16 yrs in village are married/engaged						
None	51.5	50.2	58.1	78.2	74.1	65.0
Some	42.9	39.7	31.9	12.2	2.7	10.0
Many/most	5.6	10.1	10.0	9.6	23.2	25.0
Girls < 16 yrs in village approve of marriage/engagement of girls < 16 yrs						
None	62.7*	64.7*				
Some	31.1*	24.0*				
Many/most	6.2*	11.3*				
Parents of girls < 16 yrs in village approve of marriage/ engagement of girls < 16 yrs						
None	56.5***	28.4***	15.2	15.0	32.1	25.4
Some	8.7***	24.0***	67.3	59.4	50.0	61.7
Many/most	34.8***	47.6***	17.5	25.6	17.9	12.9
People in community would shame girl/girl’s family if she did not get married < 16 yrs						
None	53.0	46.2	61.9	51.8	41.4	44.8
Some	2.3	1.3	5.6	5.0	8.6	10.9
Many/most	44.7	52.5	32.5	43.2	50.0	44.3
**School retention**						
*Perceive that* ___* in respondent’s community*						
Girls < 16 yrs in village are taken out of school before a girl is ready/completes						
None	16.2	17.7	35.4	25.2	18.2	21.4
Some	78.2	73.1	55.9	61.2	80.0	72.9
Many/most	5.6	9.2	8.7	13.6	1.8	5.7
Girls < 16 yrs in village approve of girls < 16 yrs being taken out of school before she is ready/completes						
None	66.2**	61.6**				
Some	28.8**	27.1**				
Many/most	5.0**	11.3**				
Parents of girls < 16 yrs in village approve of girls < 16 yrs being taken out of school before she is ready/completes						
None	60.5**	39.3**	6.3*	10.4*	18.7	26.1
Some	10.3**	21.6**	83.7*	69.6*	52.7	48.2
Many/most	29.2**	39.1**	10.0*	20.0*	28.6	25.7
People in community would shame a girl/girl’s family if she stayed in school (despite good marriage opportunity)						
None	51.9	47.5	61.2	68.0	60.6	60.9
Some	4.6	6.3	6.3	1.6	5.5	6.2
Many/most	43.5	46.2	32.5	30.4	33.9	32.9

*Statistically significant difference at p < 0.10

**Statistically significant difference at p < 0.05

***Statistically significant difference at p < 0.01

#### Shifts in normative expectations that girls remain in school

The evaluation indicated norms-shifting relating to the value of schooling girls and family intentions and actions that support young girls in their studies.

A grandmother from Sare Yire *“before, girls didn’t study and didn’t like school, and valued more working the land and herding animals. She showed everyone that she was…skilled in farming and small animal husbandry. Today she sees that those who study…work in offices, and those who don’t study work the fields. And studies have become important for her. All the children know [now] that studies are important and for the parent also, they intend that their children remain in school.”*

A father in Nemataba described this change in support, *“since the project arrived we don’t have our children [girls and boys] work during class days; only on days where there is no school.”*

The vignette, in which a VYA girls’ family faces financial difficulties in sending both a son and a daughter to school, revealed statistical differences between intervention and comparison groups (Table 4). Adolescent girls’ in the intervention group were more likely to perceive their peers approve (i.e., injunctive norms) that a girl should remain in school if she desired (95.0% vs. 88.7%) and to perceive their parents agree (70.2% vs. 60.9%). Likewise, caregivers in intervention villages were more likely to perceive that caregivers in their community would approve of keeping young girls in school (90.0%) than caregivers in comparison villages (80.0%). However, there was no significant differences in young girls’ or caregivers’ perceptions that many VYA girls in their village are currently enrolled in school (i.e., descriptive norms), or that there would be sanctions (e.g., shaming) for keeping girls in school. Again, there was little difference between grandmothers in intervention and comparison villages regarding the normative domains explored in the vignette.

## Discussion

This realist evaluation of the GHD approach demonstrated shift in social norms, particularly for VYA girls, in intervention villages favoring delaying girls’ marriage, preventing early pregnancy and keeping girls in school along with increased support for and action by grandmothers to support girls and their well-being related to these same outcomes. These shifts represent greater community social cohesion on girl-child issues which will benefit girls’ abilities to remain in school and avoid early marriage and pregnancy. The evaluation findings also demonstrate the alignment in attitudes, intentions, and actions in intervention villages by key stakeholder groups—VYAs, caregivers, and grandmothers—which was not seen in in comparison villages. This study took place after 18 months of project implementation although the GHD approach continued to be implemented, a time period that was too short to detect behavioral shifts.

The evaluation shows that the GHD approach based on dialogue as part of community forums and problem-solving activities to build consensus can lead to behavioral and normative shifts, and shows the power of norms-shifting interventions to improve adolescent health across interconnected outcomes. GHD especially engages grandmothers, supporting them to expand and reinforce their role as respected and valued, wise, and sought-after advisors to girls and in their families and communities. Before the intervention, formative assessments indicated that grandmothers’ traditional roles as respected advisors to VYA had diminished; community members often considered grandmothers old-fashioned and incapable of understanding young people’s hopes. GHD supported the creation of spaces to engage grandmother leaders and other grandmothers as cultural teachers in schools and as facilitators of community dialogue. The evaluation clearly shows that the strategy was sufficient to reinvigorate, update, and expand grandmothers’ roles as advocates and counselors on SRH issues, marriage, and school-going, for a more significant impact in today’s rural Senegalese society.

The GHD ToC is affirmed by these findings: using an inclusive process to involve key community actors and encourage dialogue between them through intergenerational forums will catalyze communication about new ideas and motivate individuals to act upon issues of interest to communities. The community level shift was rapid given the small-scale nature of the intervention which included several forums per month in each village. Of note also is that reflective dialogues used in different forums intentionally addressed intertwined themes of early marriage, early pregnancy, and school retention. Communities view these issues as interlinked (although they are often viewed as independent themes in adolescent programming). Accordingly, the GHD tailored its approach to such cultural realities. Finally, GHD designed their approach based on formative research, relationships within the community and the cultural realities. The program understood and built on the accepted role of grandmothers as leaders and advocates for youth well-being. Their involvement facilitated norms shifting and strengthened social cohesion. Tailoring norms-shifting interventions to the communities and cultures where they operate is important.

The realist evaluation approach is designed to explore such pathways of change and their relationship to outcomes, particularly useful for interventions operating in complex community environments and for norms-shifting interventions where causal mechanisms are not yet well understood or studied at a program level. The quantitative and qualitative findings together strongly support the program theory of change and support GMP’s previous learning studies. A longer intervention period might have allowed testing of the GHD impact on behavioral outcomes and a longitudinal study design would have helped conclusively establish whether the intervention is responsible for observed normative shifts. It is noteworthy that this is one of the first studies that uses vignettes with very young adolescents in both quantitative and qualitative research to explore social norms.

## Conclusion

Globally, programs applying norms-shifting approaches are gaining traction as important strategies to improve health. Past projects may have used norms-shifting strategies, but because they were not explicitly addressed in change theories and evaluation efforts, evidence on the effectiveness of these approaches is not fully developed. Theory-driven evaluation approaches such as Realist Evaluation are well-suited to understanding whether and how norms-shifting interventions work to achieve change as they pay explicit attention to program theory, norms shifting processes *and* outcomes, and clarify program pathways of change. They can provide guidance on good program practice to address important and interconnected VYA outcomes. Findings from this realist evaluation research help explain the social norms-behavioral change linkage, providing additional evidence that social norms-focused approaches that work via collective community consensus-building and action can lead to norms shift and early signs of behavior change.

## Data Availability

The datasets generated in this study will be made available on the USAID’s Development Data Library in early 2022.

## Abbreviations

FGD: Focus Group Discussion
GHD: Girls Holistic Development
GMP: Grandmothers Project
IDI: In Depth Interview
IRH: Institute for Reproductive Health
ToC: Theory of change
VYA: Very young adolescents
